# Amputation and mortality rates of patients undergoing upper or lower limb surgical embolectomy and their predictors

**DOI:** 10.1371/journal.pone.0279095

**Published:** 2022-12-15

**Authors:** Ákos Bérczi, Dat Tin Nguyen, Hunor Sarkadi, Balázs Bence Nyárádi, Piroska Beneda, Ádám Szőnyi, Márton Philippovich, Zoltán Szeberin, Edit Dósa

**Affiliations:** 1 Department of Interventional Radiology, Heart and Vascular Center, Semmelweis University, Budapest, Hungary; 2 Hungarian Vascular Radiology Research Group, Budapest, Hungary; 3 Department of Vascular and Endovascular Surgery, Heart and Vascular Center, Semmelweis University, Budapest, Hungary; Ataturk University Faculty of Medicine, TURKEY

## Abstract

**Purpose:**

To provide information on the outcomes of upper and lower limb surgical embolectomies and the factors influencing amputation and mortality.

**Methods:**

A retrospective, single-center analysis of 347 patients (female, N = 207; male, N = 140; median age, 76 years [interquartile range {IQR}, 63.2–82.6 years]) with acute upper or lower limb ischemia due to thromboembolism who underwent surgery between 2005 and 2019 was carried out. Patient demographics, comorbidities, medical history, the severity of acute limb ischemia (ALI), preoperative medication regimen, embolus/thrombus localization, procedural data, in-hospital complications/adverse events and their related interventions, and 30-day mortality were reviewed in electronic medical records. Statistical analysis was performed using the Mann–Whitney *U* test and Fisher’s exact test; in addition, univariate and multivariate logistic regression was conducted.

**Results:**

The embolus/thrombus was localized to the upper limb in 134 patients (38.6%) and the lower limb in 213 patients (61.4%). The median length of hospital stay was 3.8 days (IQR, 2.1–6.6 days). The in-hospital major amputation rates for the upper limb, lower limb, and total patient population were 2.2%, 14.1%, and 9.5%, respectively, and the in-hospital plus 30-day mortality rates were 4.5%, 9.4%, and 7.5%, respectively. In patients with lower limb embolectomy, the predictor of in-hospital major amputation was the time between the onset of symptoms and embolectomy (OR, 1.78), while the predictor of in-hospital plus 30-day mortality was previous stroke (OR, 7.16). In the overall patient cohort, there were two predictors of in-hospital major amputation: 1) the time between the onset of symptoms and embolectomy (OR, 1.92) and 2) compartment syndrome (OR, 3.51).

**Conclusion:**

Amputation and mortality rates after surgical embolectomies in patients with ALI are high. Patients with prolonged admission time, compartment syndrome, and history of stroke are at increased risk of limb loss or death. To avoid amputation and death, patients with ALI should undergo surgical intervention as soon as possible and receive close monitoring in the peri- and postprocedural periods.

## Introduction

Acute limb ischemia (ALI) caused by thromboembolic disease is a common vascular surgical emergency with significant morbidity and mortality [1, 2]. In ALI, a sudden decrease in tissue perfusion occurs due to the occlusion of one or more peripheral arterial segments [3]. The blockage can be secondary to many different materials, including thromboembolus, air, fat, and tumor tissue [4, 5]. The diagnosis of extensive limb thromboembolism is mainly clinical and associated with some or all of the following symptoms/complaints: pain, pallor, paresthesia, paralysis, and pulselessness [6]. The treatment of ALI is usually based on Rutherford’s classification, physical examination, and radiological imaging [6, 7]. Open surgical embolus/thrombus removal, with or without adjunctive therapy, is an accepted and widely used method for the treatment of ALI [8–12]. Unfortunately, the prognosis for ALI from thromboembolism is poor even with early intervention; the in-hospital and/or 30-day mortality rate is 0%–66% [4, 9, 13–15]. In the last 30 years, few published studies, most of which evaluated the lower limbs, have addressed the determinants of postoperative morbidity and mortality [4, 9, 13–15]. Therefore, our study aimed to provide detailed information on the success of not only lower limb but also upper limb surgical embolectomies, as well as factors influencing amputation and mortality.

## Patients and methods

### Patient selection

A retrospective, single-center analysis of 347 patients with acute upper or lower limb ischemia who underwent surgery between May 1, 2005 and December 31, 2019 was carried out. A total of 407 open and 105 purely endovascular procedures for acute upper or lower limb thromboembolic events were carried out during this period. Patients with ALI due to traumatic or iatrogenic injury, patients with a history of aortic or upper or lower limb arterial dissection or endovascular and/or open surgical repair at the site of occlusion, patients in whom the embolus/thrombus was eliminated through purely endovascularly means, and patients who could only undergo amputation were not included in this study. The study was approved by the Semmelweis University Regional and Institutional Committee of Science and Research Ethics (113/2021). All data were fully anonymized before we accessed them, and the above-mentioned ethics committee waived the requirement for informed consent.

### Diagnosis of acute limb ischemia

Diagnosis of ALI was made based on patient complaints/symptoms (sudden onset of limb pain) and physical (pallor, paresthesia, and/or paresis of the affected limb, and absent pulses distal to the site of occlusion), handheld Doppler, and/or imaging (ultrasound, computed tomography angiography, and/or digital subtraction angiography [DSA]) findings. Handheld Doppler and imaging features seen in ALI are well known and have been described elsewhere [6].

### Medication and surgery

At the time of diagnosis of ALI, patients received 5,000 IU of intravenous heparin; this dose was administered every 6 hours until the patient underwent surgery. An additional 10,000 IU of heparin was given in the operating room. Postoperative medication was determined by the aetiology of ALI and the patient’s comorbidities. If a distinction could be made between the two aetiologies (embolism versus thrombosis), long-term anticoagulation therapy with coumarin derivates was recommended for revascularization due to embolism, whereas antiplatelet and statin therapy was recommended for revascularization due to native arterial thrombosis. For revascularization, embolectomy with a Fogarty catheter was first attempted in all patients. The size of the Fogarty catheter (2–5F) varied depending on the diameter of the arteries involved. (In patients with ALI caused by an aneurysm, not only embolectomy but also aorto-aortic interposition grafting or popliteal bypass grafting was carried out.) In the upper limb, the site of surgical exploration was at the level of the origin of the deep brachial artery or, more commonly, in the cubital fossa, where the brachial artery divides into the radial and ulnar arteries. In the lower limb, the incisions were made either at the bifurcation of the common femoral artery and/or at the popliteal artery below the knee, where the crural vessels were separately accessible for catheter passage. Embolectomy was considered technically successful if good inflow and backflow was obtained from the affected arterial segment(s). Except for the last 5 years of the study period, intraoperative or postprocedural DSA was not routinely used and occurred only if deemed necessary by the operating surgeon. If DSA was performed and small residual emboli/thrombi were revealed in the runoff arteries, recombinant tissue plasminogen activator (5–10 mg; Actilyse, Boehringer Ingelheim RCV GmbH & Co KG, Wien, Austria) was administered. The recombinant tissue plasminogen activator was injected directly into the emboli/thrombi or as close to them as possible. However, if DSA demonstrated large residual emboli/thrombi or chronic stenosis in the iliofemoral or popliteal segment, further embolectomy or bypass grafting or bare metal or covered stent implantation was carried out. Stenting was also required if a flow-limiting dissection developed as a complication of the embolectomy. These adjunctive endovascular or more extensive surgical procedures (with or without fasciotomy) and amputations were done in the same or another session as the embolectomy. The indication for fasciotomy was pain, postoperative calf swelling, and the presence of paresthesia and/or paresis. During the fasciotomy, two long incisions were made on the medial and lateral sides of the calf, reaching all four muscle compartments. Patients were regularly assessed for muscle viability and necrectomies were performed if necessary. Open wounds from fasciotomy incisions were treated with smart dressings, negative pressure therapy, and gradual approximation techniques. If primary closure was not achieved within two weeks, skin grafting followed.

### Data collection

Patient demographics, risk factors for arterial disease, medical history, the severity of ALI, preoperative medication regimen, embolus/thrombus localization, procedural information, in-hospital complications/adverse events and their related interventions, and 30-day mortality were reviewed in electronic medical records. Demographic variables and risk factors for arterial disease included sex, age, smoking, hypertension, hyperlipidemia, and diabetes mellitus. The definition of each risk factor can be found in a publication by Vértes et al. [16]. Patient’s history of cardiac and ischemic cerebral diseases, aneurysm, endovascular or open surgical repair, amputation, deep vein thrombosis (DVT), pulmonary embolism (PE), hypercoagulable state, chronic obstructive pulmonary disease (COPD), and malignancy was also collected. The severity of ALI was determined according to the Rutherford classification (I, limb viable; IIa, limb marginally threatened; IIb, limb immediately threatened; and III, limb irreversibly damaged) [17]. For medication, the focus of the study was on anticoagulants, steroids, and chemotherapy. Evaluation of the localization of the embolus/thrombus was based on physical examination, handheld Doppler and imaging findings, and/or intraoperative results. Procedural information included the time between the onset of symptoms and embolectomy, the location of arterial exposure, and the type of procedure(s) executed in case of embolectomy failure. Reocclusion, distal embolization, reperfusion injury, compartment syndrome, incision site complications, acute kidney injury, myocardial infarction (MI), stroke, minor or major amputation, and death were examined as in-hospital complications/adverse events. Incision site complications were taken into account if they necessitated additional interventions, prolonged hospital stay, or both. The 30-day mortality was equivalent to death within 30 days of discharge from the hospital. Minor amputation was defined as removal of a hand or foot, or a part thereof, while amputations above the wrist and ankle joints were considered major amputations.

### Statistical analysis

Statistical analysis was performed with SPSS Statistics for Windows (Version 27.0.; IBM Corp., Armonk, NY, USA). Continuous variables were expressed as medians (interquartile ranges [IQRs]) and compared using the Mann–Whitney *U* test. Categorical data were expressed as counts (percentages) and compared using the Fisher’s exact test. Univariate logistic regression was applied to calculate odds ratios (ORs). Variables that were statistically significant in the univariate model were additionally used in a multivariate logistic regression analysis adjusted for sex and age. All statistical tests were two-tailed. The threshold of statistical significance was P<0.05.

## Results

### Patient characteristics

Of the 347 patients, 207 (59.7%) were female, 140 (40.3%) were male, and the median age was 76 years (IQR, 63.2–82.6 years). One hundred and forty-one patients (40.6%) were active smokers and 253 patients (72.9%), 86 patients (24.8%), and 92 patients (26.5%) had known hypertension, hyperlipidemia, and diabetes mellitus, respectively. (Table 1) The medical history of the patients can be seen in Table 2. ALI was categorized as stage I in four patients (1.2%), stage IIa in 92 patients (26.5%), and stage IIb in 251 patients (72.3%). (Table 1) At the time of embolization/thrombosis, 78 patients (22.5%) received anticoagulant therapy, seven patients (2%) received steroid therapy, and 52 patients (15%) received chemotherapy. (Table 1) The cause of anticoagulation was atrial fibrillation (AF) in 52 cases, other cardiac diseases in 15 cases, ischemic stroke in six cases, DVT and/or PE in three cases, and hypercoagulable state in two cases. Despite the medical recommendation, 15 patients did not take the oral anticoagulant.

**Table 1:** Patient demographics, risk factors for arterial disease, the severity of acute limb ischemia, and preoperative medication regimen.

Patient demographics, risk factors, severity of ALI, and preoperative medication regimen	All patients (N = 347)	Patients with upper limb surgical embolectomy (N = 134)	Patients with lower limb surgical embolectomy (N = 213)	P-value
Patient demographics				
Female, N (%)	207 (59.7)	88 (65.7)	119 (55.9)	0.070
Male, N (%)	140 (40.3)	46 (34.3)	94 (44.1)	0.070
Age (years), median (IQR)	76 (63.2–82.6)	76 (64–83.5)	76.2 (61.6–82.2)	0.509
Risk factors				
Smoking, N (%)	141 (40.6)	50 (37.3)	91 (42.7)	0.318
Hypertension, N (%)	253 (72.9)	100 (74.6)	153 (71.8)	0.568
Hyperlipidemia, N (%)	86 (24.8)	31 (23.1)	55 (25.8)	0.572
Diabetes mellitus, N (%)	92 (26.5)	38 (28.4)	54 (25.4)	0.537
Severity of ALI				
Stage I, N (%)	4 (1.2)	0 (0)	4 (1.9)	0.162
Stage IIa, N (%)	92 (26.5)	46 (34.3)	46 (21.6)	0.009
Stage IIb, N (%)	251 (72.3)	88 (65.7)	163 (76.5)	0.036
Preoperative medication regimen				
Anticoagulant therapy, N (%)	78 (22.5)	30 (22.4)	48 (22.5)	0.974
Steroid therapy, N (%)	7 (2)	3 (2.2)	4 (1.9)	0.817
Chemotherapy, N (%)	52 (15)	24 (17.9)	28 (13.1)	0.226

*ALI*, Acute limb ischemia; *IQR*, interquartile range.

**Table 2:** Patient medical history.

Pre-existing diseases/abnormalities and previous invasive vascular procedures, N (%)	All patients (N = 347)	Patients with upper limb surgical embolectomy (N = 134)	Patients with lower limb surgical embolectomy (N = 213)	P-value
Cardiac disease				
Arrhythmia	90 (25.9)	30 (22.4)	60 (28.2)	0.232
AF	67 (19.3)	25 (18.7)	42 (19.7)	0.807
Coronary artery disease	59 (17)	19 (14.2)	40 (18.8)	0.267
Valvular disease	8 (2.3)	3 (2.2)	5 (2.3)	1.000
Heart failure	3 (0.9)	1 (0.7)	2 (0.9)	1.000
Ischemic cerebral disease				
Amaurosis fugax, TIA	19 (5.5)	7 (5.2)	12 (5.6)	0.870
Stroke	14 (4)	7 (5.2)	7 (3.3)	0.372
Aneurysm				
Aortic aneurysm	1 (0.3)	0 (0)	1 (0.5)	1.000
Peripheral aneurysm	2 (0.6)	1 (0.7)	1 (0.5)	1.000
Peripheral endovascular therapy*	83 (23.9)	35 (26.1)	48 (22.5)	0.167
Peripheral open surgical repair*	62 (17.9)	22 (16.4)	40 (18.8)	0.106
Amputation				
Minor amputation	5 (1.4)	0 (0)	5 (2.3)	0.161
Major amputation	3 (0.9)	0 (0)	3 (1.4)	0.287
DVT and/or PE	3 (0.9)	1 (0.7)	2 (0.9)	1.000
Hypercoagulable state	2 (0.6)	1 (0.7)	1 (0.5)	1.000
COPD	7 (2)	2 (1.5)	5 (2.3)	0.711
Malignancy	72 (20.7)	30 (22.4)	42 (19.7)	0.588

*AF*, Atrial fibrillation; *COPD*, chronic obstructive pulmonary disease; *DVT*, deep vein thrombosis; *PE*, pulmonary embolism; *TIA*, transient ischemic attack.

*Unrelated to the site of acute arterial occlusion.

### Embolus/thrombus localization

The embolus/thrombus was localized to the upper limb in 134 patients (38.6%) and the lower limb in 213 patients (61.4%). Simultaneous upper and lower limb involvement did not occur in any of the patients. No patient had bilateral embolization/thrombosis on the upper limb, while 18 patients (5.2%) had bilateral embolization/thrombosis on the lower limb.

In the upper limb, the following arteries were affected: the subclavian artery in 11 cases, the axillary artery in 11 cases, the brachial artery in 118 cases, the radial artery in 47 cases, the ulnar artery in 57 cases, and the interosseous artery in 12 cases. The involvement of more than one artery was seen in 69 patients.

In the lower limb, the following arteries were affected unilaterally or bilaterally: the common iliac artery in four cases, the external iliac artery in 31 cases, the common femoral artery in 81 cases, the deep femoral artery in 83 cases, the superficial femoral artery in 89 cases, the popliteal artery in 112 cases, the anterior tibial artery in 37 cases, the tibioperoneal trunk in 26 cases, the posterior tibial artery in 11 cases, and the peroneal artery in seven cases. The involvement of more than one artery was observed in 140 patients.

### Procedural information

In the upper limb, the time between the onset of symptoms and embolectomy was less than 24 hours in 98 patients (73.1%), 1 to 7 days in 28 patients (20.9%), and more than 1 week in eight patients (6%). The site of surgical exploration was the axillary artery in 20 cases (14.9%), the brachial artery in 104 cases (77.6%), and both arteries in 10 cases (7.5%). Embolectomy was technically successful in 118 patients (88.1%). Fifteen patients received additional invasive treatment (intra-arterial thrombolytic therapy with or without further embolectomy, N = 5; innominate artery stenting, N = 2; and subclavian artery stenting, N = 8). Each patient had adjunctive therapy only once. The median time between embolectomy and adjunctive therapy was 4 hours (IQR, 0.5–12.5 hours). Concomitant amputation with embolectomy was unavoidable in one case (major amputation, N = 1). Neither stage IIb ALI (25/36 versus 63/98, P = 0.683) nor failed embolus/thrombus removal (5/36 versus 10/98, P = 0.546) was significantly more common in patients who underwent embolectomy after 24 hours.

**Fig 1:**
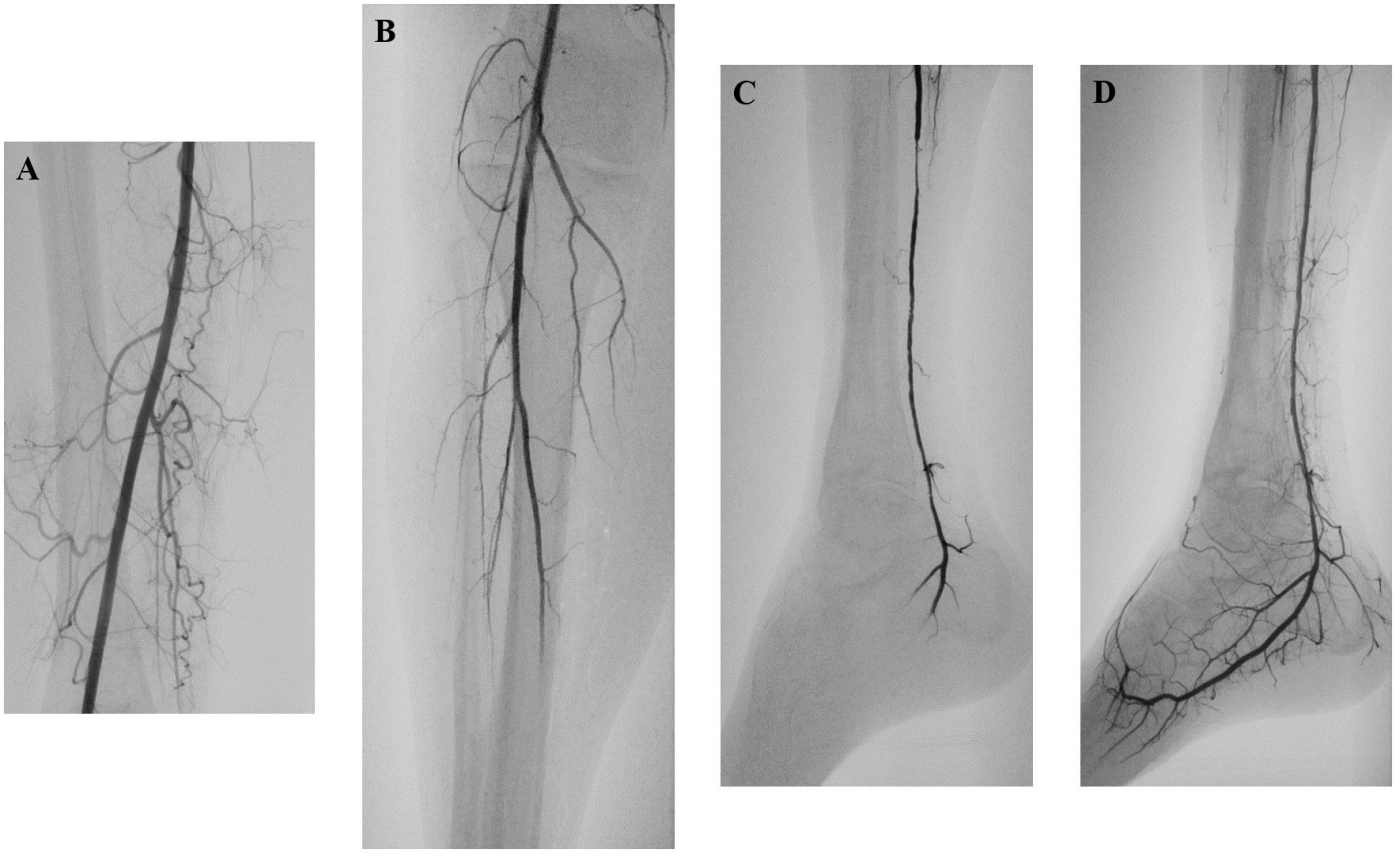
Right posterior tibial artery embolectomy combined with intra-arterial thrombolytic therapy. (A) No evidence of atherosclerosis or embolism in the superficial femoral artery. (B) No evidence of atherosclerosis or embolism in the popliteal artery. Chronic occlusion of the distal two-thirds of the anterior tibial artery and peroneal artery as a result of a vascular injury sustained during excision of a sarcoma 5 years ago. Acute occlusion of the distal two-thirds of the posterior tibial artery by embolism. (C) Contrast material filling in the posterior tibial artery after removal of the embolus with a Fogarty catheter. No contrast material filling in the plantar arch. (D) Perfect morphology of the posterior tibial artery and plantar arch on angiography 24 hours after intra-arterial administration of 10 mg recombinant tissue plasminogen activator directly into the posterior tibial artery.

In the lower limb, the time between the onset of symptoms and embolectomy was less than 24 hours in 138 patients (64.8%), 1 to 7 days in 51 patients (23.9%), and more than 1 week in 24 patients (11.3%). The site of surgical exploration was one of the common femoral arteries in 84 cases (39.4%), one of the popliteal arteries in 80 cases (37.6%), the equilateral common femoral and popliteal arteries in 15 cases (7%), the common femoral artery on one side and the popliteal artery on the opposite side in seven cases (3.3%), the common femoral artery on both sides in 17 cases (8%), and the popliteal artery on both sides in 10 cases (4.7%). Embolectomy was technically successful in 176 patients (82.6%). Twenty-seven patients received additional invasive treatment (intra-arterial thrombolytic therapy (Fig 1) with or without further embolectomy, N = 15; common iliac or external iliac artery stenting, N = 5; superficial femoral artery stenting, N = 5; femoral patch plasty, N = 4; aorto-aortic interposition grafting, N = 2; and/or femoropopliteal bypass grafting, N = 4). Eight patients had adjunctive therapy twice. For those who received adjunctive therapy only once, the median time between embolectomy and adjunctive therapy was 7 hours (IQR, 2.8–10 hours), while for those who received adjunctive therapy twice, the median time between embolectomy and second adjunctive therapy was 12.5 hours (IQR, 9.5–18.8 hours). Fasciotomy was required in 12 cases. Concomitant amputation with embolectomy was unavoidable in 10 cases (major amputation, N = 10). Neither stage IIb ALI (58/75 versus 105/138, P = 0.867) nor failed embolus/thrombus removal (14/75 versus 13/138, P = 0.083) was significantly more common in patients who underwent embolectomy after 24 hours.

### In-hospital complications/adverse events and 30-day mortality

The median length of hospital stay was 3.8 days (IQR, 2.1–6.6 days). The presumed cause of embolization/thrombosis was a pre-existing disease in 204 patients (58.8%) and a newly discovered disease/condition in 39 patients (11.2%; AF, N = 20; other cardiac diseases, N = 15; aortic aneurysm, N = 1; and popliteal aneurysm, N = 3). In 104 cases (30%), the cause was not found.

In-hospital complications/adverse events are summarized in Table 3. The in-hospital reocclusion rate was 5.2%, the acute kidney injury rate was 1.2%, the MI rate was 1.2%, the stroke rate was 1.2%, the major amputation rate was 9.5%, and the mortality rate was 2.6%. Two out of three patients who had upper limb major amputation and two out of 30 patients who had lower limb major amputation died during their hospital stay. Reperfusion injury occurred in 25 patients (7.2%) and compartment syndrome in 20 patients (5.8%). Compartment syndrome was significantly more common in those who underwent surgery after 24 hours (8/111 versus 12/236, P = 0.473) and not significantly more common in those who received adjunctive therapy (5/42 versus 15/305, P = 0.079). Reperfusion injury led to severe metabolic abnormalities (hyperkalemia, metabolic acidosis, and rhabdomyolysis/myoglobinuria) in three patients and loss of limb in two patients. The 30-day mortality rate was 4.9% (upper limb, N = 3 [2.2%] and lower limb, N = 14 [6.6%]). The cause of in-hospital death was cardiorespiratory insufficiency in four cases, cardiogenic shock in one case, end-stage heart failure in one case, MI in one case, stroke in one case, and sepsis in one case. The cause of 30-day death was MI in three cases, stroke in three cases, gastrointestinal bleeding in two cases, and unknown in nine cases.

**Table 3:** In-hospital complications/adverse events.

Complications/adverse events, N (%)	All patients (N = 347)	Patients with upper limb surgical embolectomy (N = 134)	Patients with lower limb surgical embolectomy (N = 213)	P-value
Reocclusion	18 (5.2)	9 (6.7)	9 (4.2)	0.197
Distal embolization	12 (3.5)	3 (2.2)	9 (4.2)	0.383
Reperfusion injury	25 (7.2)	5 (3.7)	20 (9.4)	0.055
Compartment syndrome	20 (5.8)	0 (0)	20 (9.4)	<0.001
Incision site complications	23 (6.6)	9 (6.7)	14 (6.6)	0.958
Acute kidney injury	4 (1.2)	0 (0)	4 (1.9)	0.162
MI	4 (1.2)	1 (0.7)	3 (1.4)	1.000
Stroke	4 (1.2)	2 (1.5)	2 (0.9)	0.562
Amputation	34 (9.8)	3 (2.2)	31 (14.6)	<0.001
Minor amputation	1 (0.3)	0 (0)	1 (0.5)	1.000
Major amputation	33 (9.5)	3 (2.2)	30 (14.1)	<0.001
Death	9 (2.6)	3 (2.2)	6 (2.8)	1.000

*MI*, Myocardial infarction.

### Predictors of in-hospital major amputation and in-hospital plus 30-day mortality

Due to the statistically low number of cases, predictive factors could only be examined in relation to lower limb embolectomies and embolectomies in the entire patient population; of the predictors, only those for in-hospital major amputation and in-hospital plus 30-day mortality were determined. The predictive role of all the parameters described in the Data Collection subsection has been evaluated. The parameters found to be significant in univariate and multivariate analyses are presented in Tables 4 and 5. In patients with lower limb embolectomy, the time between the onset of symptoms and embolectomy was a predictor of in-hospital major amputation (OR, 1.78), while previous stroke was a predictor of mortality (OR, 7.16). (Table 5) In patients with upper or lower limb embolectomy, two predictive factors were identified for in-hospital major amputation: 1) the time between the onset of symptoms and embolectomy (OR, 1.92) and 2) compartment syndrome (OR, 3.51) (Table 5).

**Table 4:** Significant predictors of in-hospital major amputation and in-hospital plus 30-day mortality—Univariate analysis.

Parameters	Odds ratio	Confidence interval	P-value
Major amputation in patients with lower limb embolectomy			
Time between the onset of symptoms and embolectomy	1.67	1.01–2.78	0.047
Compartment syndrome	3.98	1.44–11	0.008
Major amputation in patients with upper or lower limb embolectomy			
Diabetes mellitus	2.23	1.07–4.66	0.033
Previous minor amputation	6.69	1.08–41.56	0.041
Lower limb involvement	7.16	2.14–23.95	0.001
Time between the onset of symptoms and embolectomy	1.67	1.01–2.78	0.047
Compartment syndrome	6.23	2.29–16.99	<0.001
Mortality in patients with lower limb embolectomy			
Previous stroke	8.34	1.72–40.36	0.008
Mortality in patients with upper or lower limb embolectomy			
Malignancy	2.61	1.13–6.03	0.025
Chemotherapy	2.80	1.15–6.82	0.024

**Table 5:** Significant predictors of in-hospital major amputation and in-hospital plus 30-day mortality—Multivariate analysis.

Parameters	Odds ratio	Confidence interval	P-value
Major amputation in patients with lower limb embolectomy			
Time between the onset of symptoms and embolectomy	1.78	1.05–3.01	0.033
Major amputation in patients with upper or lower limb embolectomy			
Time between the onset of symptoms and embolectomy	1.92	1.10–3.34	0.022
Compartment syndrome	3.51	1.17–10.52	0.025
Mortality in patients with lower limb embolectomy			
Previous stroke	7.16	1.43–36.01	0.017

## Discussion

In this single-center study, the results of upper and lower limb surgical embolectomies were analyzed in a large cohort. There are few complex (comprehensive) studies in the literature on surgical embolectomy in patients with ALI. Only two relevant publications were found for upper limb embolectomies. One is a retrospective evaluation of 405 patients in the Danish national registry [18], while the other is a meta-analysis [13]. The purpose of the latter was to investigate the relationship between AF and peripheral thromboembolism in terms of incidence, risk factors, risk-modifying drugs, and prognosis [13]. In the context of lower limb embolectomies, three recent retrospective studies should be highlighted. Kempe et al. [9] conducted a single-center study in 170 patients, while the other two studies were multicenter and involved 136 and 1749 patients, respectively [14, 15]. Apart from our study, there is only one study in total that includes both upper and lower limb embolectomies: a single-center retrospective Turkish study in 822 patients [4].

The short-term outcomes of surgical limb embolectomies can be characterized by amputation and mortality rates. The prevalence and predictive parameters of amputation and mortality are discussed below. However, comparison of the rates is hampered by the fact that amputation and mortality time intervals often differ from study to study, and neither the type of amputation nor the time intervals are (accurately) defined. In our study, in-hospital amputation rates for the upper limb, lower limb, and the entire patient population were 2.2%, 14.6%, and 9.8%, respectively. The incidence of upper limb amputations in Andersen’s [13] meta-analysis ranged from 0% to 8% (based on 22 studies conducted between 1932–1962 and 1993–2017), while Dag et al. [4] demonstrated an incidence of 2.8%, which is very similar to our result. The lower limb amputation rates in the two relevant publications were 16% (major, 15% and minor, 1%) [9] and 10.8% [4], while our in-hospital rate was between the two. Data on amputation of the lower limbs were not provided by the other two major studies [14, 15]. The combined amputation rate of upper and lower limb embolectomies is available in only one study; Dag et al. [4] mentioned an amputation incidence of 13.6%, which is slightly higher than the incidence we obtained. Kempe et al. [9] was the only study to state that the amputation rate was given for a period of 90 days. None of the other publications specified the time interval to which the amputation rate applied.

In this study, the in-hospital mortality rates for the upper limb, lower limb, and the entire patient population were 2.2%, 2.8%, and 2.6%, respectively, while the 30-day mortality rates for the upper limb, lower limb, and the entire patient population were 2.2%, 6.6%, and 4.9%, respectively. Andersen’s [13] meta-analysis revealed a mortality rate of 0%–66% (based on 15 studies) for upper limb embolectomies. Previous publications indicate that the mortality rate for lower limb embolectomies is 6.9%–18% [9, 14, 15]. These mortality rates refer to in-hospital and/or 30-day mortality [9, 13–15]. The combined mortality rate of upper and lower limb embolectomies is available in only one study in which the authors mention a 2% “early” postoperative mortality [4]. However, the authors did not define what was meant by “early”. The mortality rates reported in our study are among the lowest of those published.

In our study, predictive factors could only be examined in relation to lower limb embolectomies and embolectomies in the entire patient population; of the predictors, only those for in-hospital major amputation and in-hospital plus 30-day mortality were determined. We have shown that the time between the onset of symptoms and embolectomy is a predictor of lower limb major amputation, while the time between the onset of symptoms and embolectomy and compartment syndrome are predictors of all (upper plus lower limb) major amputations. Others have identified female sex [13], previous vascular surgical procedure [9], presence of gangrene [9], need for fasciotomy [9], and re-embolectomy [4] as predictors of amputation. Dag et al. [4], like us, emphasize the importance of admission time, as a duration longer than 6 hours—presumably through a complex cascade induced by ischemia and hypoxia in the affected limb—significantly increases the risk of amputation (OR, 40.3).

In this study, history of stroke proved to be a predictor of in-hospital plus 30-day mortality in patients with lower limb embolectomy. Information on predictors of mortality in the literature is limited to lower limb embolectomies. Two studies report predictive factors for mortality. One considers previous vascular surgical procedure and concurrent stroke [9], while the other considers male sex, functional dependence, COPD, chronic heart failure, recent angina/MI, chronic renal insufficiency, and steroid use as predictors of mortality [15] in addition to age [9, 15]. Based on these findings, patients with comorbidities that potentiate the process of atherosclerosis or patients who already have manifest vascular disease appear to be a special risk group in terms of mortality.

The limitations of this study may have influenced our results. First, patients were retrospectively enrolled. Second, it is not always possible to clearly distinguish an acute embolic case from an acute thrombotic case, so these cases were analyzed as a group rather than separately. Third, unlike mortality, for amputations, only in-hospital data could be retrieved from our database.

In conclusion, amputation and mortality rates after surgical embolectomies in patients with ALI are high. Patients with prolonged admission time, compartment syndrome, and history of stroke are at increased risk of limb loss or death. To avoid amputation and death, patients with ALI should undergo surgical intervention as soon as possible and receive close monitoring in the peri- and postprocedural periods.

## Supporting information

S1 Table Evaluated parameters. (XLS)
